# The Movesense Medical Sensor Chest Belt Device as Single Channel ECG for RR Interval Detection and HRV Analysis during Resting State and Incremental Exercise: A Cross-Sectional Validation Study

**DOI:** 10.3390/s22052032

**Published:** 2022-03-05

**Authors:** Bruce Rogers, Marcelle Schaffarczyk, Martina Clauß, Laurent Mourot, Thomas Gronwald

**Affiliations:** 1 Department of Internal Medicine, College of Medicine, University of Central Florida, Orlando, FL 32827, USA; 2 Department Sports and Exercise Medicine, Institute of Human Movement Science, University of Hamburg, 20148 Hamburg, Germany; marcelle.schaffarczyk@uni-hamburg.de; 3 Institute of Movement and Trainings Science in Sport, Faculty of Sport Science, Leipzig University, 04109 Leipzig, Germany; klausm@rz.uni-leipzig.de; 4 EA3920 Pronostic Factors and Regulatory Factors of Cardiac and Vascular Pathologies, Exercise Performance Health Innovation (EPHI) Plaptform, University of Bourgogne Franche-Comté, 25000 Besançon, France; laurent.mourot@univ-fcomte.fr; 5 Division for Physical Education, National Research Tomsk Polytechnic University, 634040 Tomsk, Russia; 6 Institute of Interdisciplinary Exercise Science and Sports Medicine, MSH Medical School Hamburg, 20457 Hamburg, Germany; thomas.gronwald@medicalschool-hamburg.de

**Keywords:** heart rate variability, wearable, medical device, health care, exercise

## Abstract

The value of heart rate variability (HRV) in the fields of health, disease, and exercise science has been established through numerous investigations. The typical mobile-based HRV device simply records interbeat intervals, without differentiation between noise or arrythmia as can be done with an electrocardiogram (ECG). The intent of this report is to validate a new single channel ECG device, the Movesense Medical sensor, against a conventional 12 channel ECG. A heterogeneous group of 21 participants performed an incremental cycling ramp to failure with measurements of HRV, before (PRE), during (EX), and after (POST). Results showed excellent correlations between devices for linear indexes with Pearson’s r between 0.98 to 1.0 for meanRR, SDNN, RMSSD, and 0.95 to 0.97 for the non-linear index DFA a1 during PRE, EX, and POST. There was no significant difference in device specific meanRR during PRE and POST. Bland–Altman analysis showed high agreement between devices (PRE and POST: meanRR bias of 0.0 and 0.4 ms, LOA of 1.9 to −1.8 ms and 2.3 to −1.5; EX: meanRR bias of 11.2 to 6.0 ms; LOA of 29.8 to −7.4 ms during low intensity exercise and 8.5 to 3.5 ms during high intensity exercise). The Movesense Medical device can be used in lieu of a reference ECG for the calculation of HRV with the potential to differentiate noise from atrial fibrillation and represents a significant advance in both a HR and HRV recording device in a chest belt form factor for lab-based or remote field-application.

## 1. Introduction

The biosignal of heart rate (HR) variability (HRV) signifying cardiac interbeat interval variation is a promising candidate as a non-invasive marker for the exploration of cardiac autonomic function, with applications both in remote, local laboratory, and clinical settings [1,2,3]. In general, the variability in cardiac interbeat intervals is the result of the interaction of multiple regulatory mechanisms that operate at different time scales [4]. This includes long-term mechanisms (e.g., circadian rhythms) and short-term processes integrating the autonomic, cardiovascular, and respiratory systems [5,6]. Consequently, HRV possesses a broad range of applications in health and disease. Examples include biofeedback interventions in chronic disease management [7], the assessment of cognitive function, impairment and aging [8,9], psychological states in health and disease, including posttraumatic stress disorder or psychiatric disorders such as depression or anxiety [10,11,12], pain disorders [13,14], viral infections [15,16], as a prognostic factor for cancer survival [17], endurance exercise and training prescription, and the effect of exercise and training interventions in both resting states as well as during dynamic exercise and post-exercise recovery [2,4,18,19,20,21]. 

As another example, lower HRV has been associated with poor prognosis in specific clinical conditions such as cardiovascular diseases, specifically risk stratification, and mortality prediction [22,23,24]. Additionally, age, as a non-modifiable factor, also influences the autonomic nervous system regulation of the heart, with decreasing HRV during one’s lifespan [25]. In contrast, regular behavior like physical activity and/or exercise, balanced diet, and/or mindfulness have been shown to increase HRV, particularly highlighted in measures of vagal function [26]. Holistically viewed, higher HRV has been associated with better health, psycho-physiological capacity, and performance status, since specific measures of HRV showed high sensitivity to alterations in acute and chronic status of fitness and fatigue, including numerous personal and environmental influencing factors [2,18,27,28,29,30,31,32]. Thus, the analysis of HRV offers insights into humoral, neural, and neurovisceral processes in health, disease, and behavioral patterns, but has yet to be fully potentiated in the digital age [3,33]. 

Because of the high inter- and intraindividual variation in cardiac interbeat intervals, the task force standards for HRV measurement, physiological interpretation, and clinical use [34] and its update [1] suggest that an evaluation of commercially available wearable devices is required to ensure the validity of RR interval measurement for precise interpretation of HRV measures, even though a required level of agreement is not specified [35]. A recent systematic review and meta-analysis from Dobbs et al. [36] demonstrates that HRV measurements acquired from portable devices demonstrate a small amount of absolute error compared to “gold standard” lab-based ECG. The authors concluded that this small error in accuracy is acceptable when considering the improved usability and user application compliance of HRV measurements and analysis acquired through low-cost portable devices for remote application in health and disease. The analysis and recommendations for determining validity of consumer wearable HR(V) devices by Mühlen et al. [35] also stated the necessity of validation studies of new devices in the specific field of application (remote, laboratory, or clinical).

**Figure 1:**
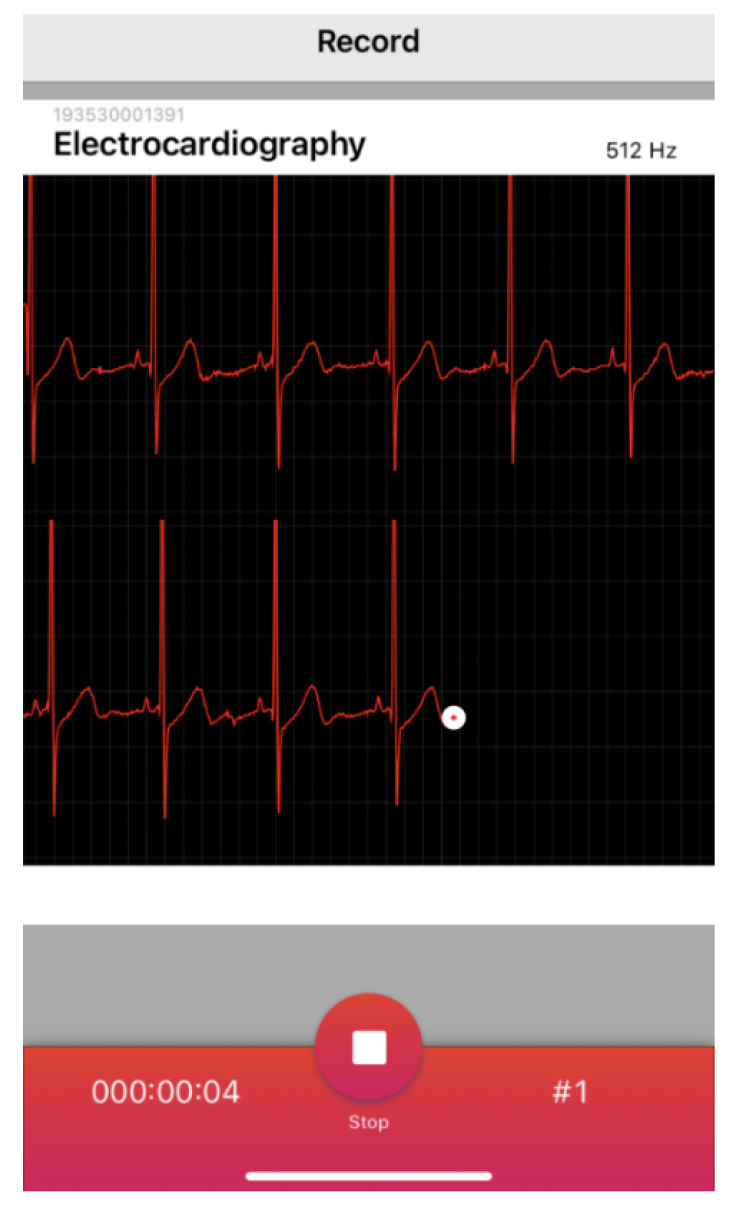
Real-time view of single channel ECG recorded (max. sampling rate: 512 Hz) with the chest belt sensor Movesense Medical displayed in the data logger app Movesense Showcase, here for iOS operating system.

For collecting real-world data during settings such as the resting state, physical activity, and exercise, there are only a few validated devices marketed for the detection of RR intervals and analysis of HRV measures as chest strap units [35], such as the H series from Polar Electro Oy (Kempele, Finland) with its current model H10. The importance of obtaining precise HR time series data during orthostatic changes (e.g., lying to sitting, sitting to standing) or during motion (physical activity or exercise demands) is especially important in the areas of health and medical care. Hence, it is desirable to be able to describe and detect artifacts for both correction and identification of potential serious arrythmias. Therefore, a significant downside of wearable technology in the current market is the inability to differentiate the loss of data quality from noise, artifact, or arrythmia [35]. In this regard, the Movesense Medical module (Movesense, Vantaa, Finland), a new chest belt sensor, analyzes data as a single channel ECG. This in turn will provide insights for better artefact detection, identification of cardiac arrythmias, and assessment of raw data quality. Additionally, a data logger app with real-time viewing mode is available for measuring RR intervals and subsequent HRV analysis (see Figure 1). The Movesense Medical module is registered as a class IIa medical device accessory, in conformity with the EU medical device directive 93/42/EEC for single channel ECG, HR, and RR interval detection (as well as providing measures of motion via acceleration, gyro, magnetometer). It is capable of ECG sample rates of 125 up to 512 Hz and possesses on board data storage (6 min at 250 Hz).

Currently, there are no published data assessing the validity of this device compared to an ECG system during rest, physical activity, and/or exercise conditions. Therefore, the aim of the present study was to evaluate the agreement of the Movesense Medical sensor chest belt device as single channel ECG compared to a 12 channel ECG device for RR interval detection, as well as selected HRV measures during rest, incremental cycling exercise, and post-exercise recovery.

## 2. Materials and Methods

### 2.1. Participants

A heterogeneous group of 21 physically active volunteers were recruited (men: *n* = 12, age: 43 ± 13 years, height: 178.2 ± 8.2 cm, body weight: 83.4 ± 13.9 kg; women: *n* = 9, age: 35 ± 11 years, height: 169.4 ± 4.4 cm, body weight: 66.4 ± 10.1 kg). The call for studies was made by word of mouth as well as via the internet and appealed to women and men of any fitness level without previous medical history, current medications, or recent illness. Participants were asked to abstain from caffeine, alcohol, tobacco, and vigorous exercise 24 h before testing. Each participant delivered written informed consent. Ethical approval for the study was obtained by the University of Hamburg, Department of Psychology and Movement Science, Germany (reference no.: 2021_400), and is in accordance with the principles of the Declaration of Helsinki. 

### 2.2. Exercise Protocol and Data Acquisition

**Figure 2:**
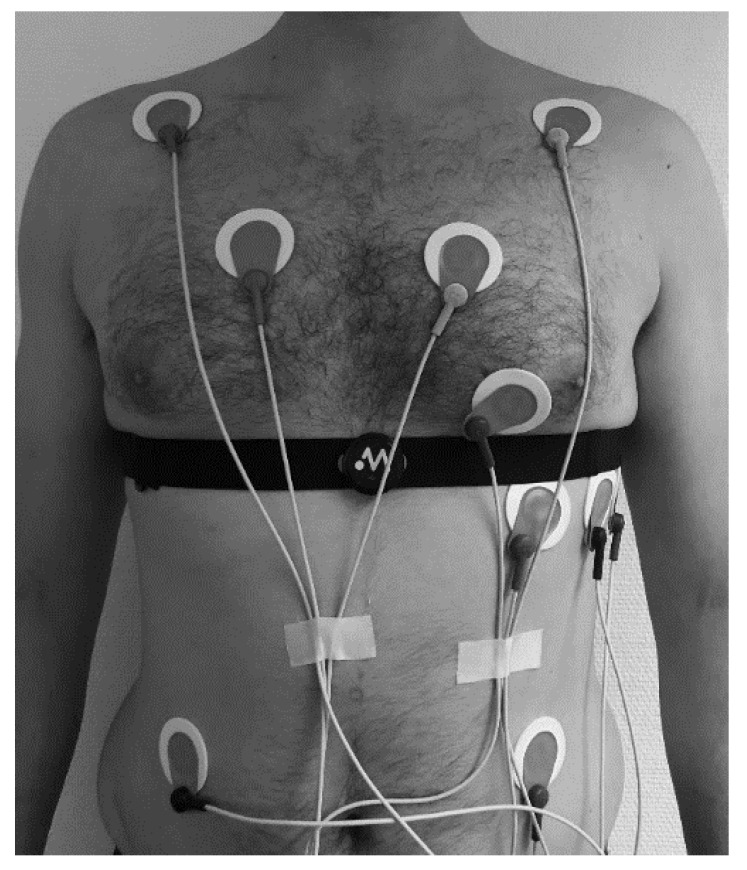
Placement of 12 channel ECG electrodes and Movesense Medical sensor single channel ECG with chest belt on one participant.

Participants performed an incremental ramp protocol until voluntary exhaustion on a mechanically braked cycle (Ergoselect 4 SN, Ergoline GmbG, Bitz, Germany). After 3 min at 50 Watts, the cycling power increased by 1 watt every 3.6 s (equivalent to 50 Watts/3 min). Prior (PRE; after a period of habituation and session preparation) and post (POST) the exercise test (after 5 min of unloaded padelling), a 3-min supine rest condition measurement interval was applied. ECG was continuously recorded wearing two devices simultaneously: (1) 12 channel ECG CardioPart 12 Blue (AMEDTEC Medizintechnik Aue GmbH, Aue, Germany; sampling rate: 500 Hz; desktop software: AMEDTEC ECGpro version 5.10.002), and (2) Movesense Medical sensor (firmware version 2.0.99) single channel ECG with chest belt (Movesense, Vantaa, Finland; sampling rate: 512 Hz; app software: Movesense Showcase version 1.0.9). Figure 2 shows the electrode placement of both devices on one participant. Gas exchange kinetics were recorded with a metabolic analyzer (Quark cpet, module A-67-100-02, COSMED Deutschland GmbH, Fridolfing, Germany; desktop software: Omnia version 1.6.5). The protocol was terminated when the subject could not hold the predetermined cadence (60 rpm), or due to self-determination. Exhaustion was assumed when the following criteria were fulfilled: (A) heart rate >90% of the maximum predicted heart rate (prediction model according to Tanaka et al., [37]: 208 − (0.7 × age) and (B) respiratory quotient >1.1. Maximum oxygen uptake (VO_2MAX_) and maximum HR (HR_MAX_) was defined as the average V˙O_2_ and HR over the last 30 s of the test. 

### 2.3. Data Processing

12 channel ECG data .txt files (converted from exported .xml files) and single channel ECG .csv files (exported from the Movesense Showcase app) were imported into Kubios HRV Software Version 3.5.0 (Biosignal Analysis and Medical Imaging Group, Department of Physics, University of Kuopio, Kuopio, Finland, Tarvainen et al. [38]). For the 12 channel ECG, lead 2 (II) was used as the comparable lead to the chest belt device [39]. Preprocessing settings were set to the default values, including the RR detrending method, which was kept at “smoothness priors” (Lambda = 500). The RR series was then corrected by the Kubios HRV “automatic method” [40] and selected parameters of HRV exported as .csv files for further analysis (meanRR: mean of RR intervals, in ms; SDNN: standard deviation of RR intervals, in ms; RMSSD: square root of the mean squared differences of the successive RR intervals, in ms; DFA a1: short-term scaling exponent of Detrended Fluctuation Analysis). 

In this regard, SDNN reflects the total variability, which is the sympathetic and parasympathetic contribution of the autonomic nervous system on the sinus node [41], and RMSSD reflects the parasympathetic influence [42], both as time domain measures of HRV with a broad range of applications [2,18]. DFA a1 is a non-linear index of HRV representing fractal correlation properties of cardiac interbeat intervals caused by physiological processes [43,44]. This index has been used for analysis of age effects [45,46], prognosis of mortality and cardiovascular risk stratification [24,47,48,49,50], and assessment of systemic internal load during endurance exercise [32,51,52,53], and appears as a relevant non-linear parameter for short-term analyses. DFA a1 window width was set to 4 ≤ *n* ≤ 16 beats [44]. During rest conditions a 2-min time window (00:30–02:30 min:s) was chosen for the analysis [54,55,56]. During the incremental exercise, time-varying analysis was adjusted to a 2-min window width and 20-s grid interval for the moving window. The exported .csv files contained meanRR, SDNN, RMSSD, and DFA a1 values recalculated every 20 s. Artifact levels measured by Kubios HRV were below 5%.

### 2.4. Statistics

Statistical analysis was performed for the tested variables using standard methods for the calculation of means, medians, and standard deviations (SD). Normal distribution of data was checked by Shapiro-Wilk testing and visual inspection of data histograms.

To determine the agreement of the analyzed variables of the two devices during PRE, incremental exercise and POST, linear regression, Pearson’s r correlation coefficient, coefficient of determination (R^2^), standard error of estimate (SEE), and Bland–Altman plots with limits of agreement (LOA) was used [57]. The size of Pearson’s r correlations was evaluated as follows; 0.3 ≤ r < 0.5 low, 0.6 ≤ r < 0.8 moderate, and r ≥ 0.8 high [58]. Estimates of the median difference were calculated using the Hodges Lehmann shift method along with Wilcoxon testing of paired groups in view of possible outlier values [59,60]. Agreement between groups was assessed by Bland–Altman analysis, but if proportional bias was detected, regression-based calculation of mean differences and limits of agreement were performed [61,62]. Bland–Altman mean differences for data comparisons was expressed as either absolute difference (ms) or percentage bias (difference/mean × 100). Paired t testing was used for comparison of the resting state measurement intervals PRE and POST the incremental exercise test. In addition, effect sizes were calculated [63]. The interpretation of effect sizes is based on Cohen’s thresholds (small effect = 0.20, medium effect = 0.50, large effect = 0.80; [64]). For all tests, the statistical significance was accepted as *p* ≤ 0.05. Analysis was performed using Microsoft Excel 365 with Real Statistics Resource Pack software (Release 6.8) and Analyse-it software (Version 5.66).

## 3. Results

The participants reached maximal power (P_MAX_) values of 260 ± 53 watts, a V˙O_2MAX_ of 40.3 ± 7.9 mL/kg/min, and a HR_MAX_ of 176 ± 13 bpm. Five participants were excluded from exercise analysis due to artefacts >5% resulting from both atrial and ventricular ectopic beats and 3 participants from resting POST measurement due to excess noise in the reference ECG, resulting in artefact >5%. 

### 3.1. PRE and POST Analysis

**Figure 3:**
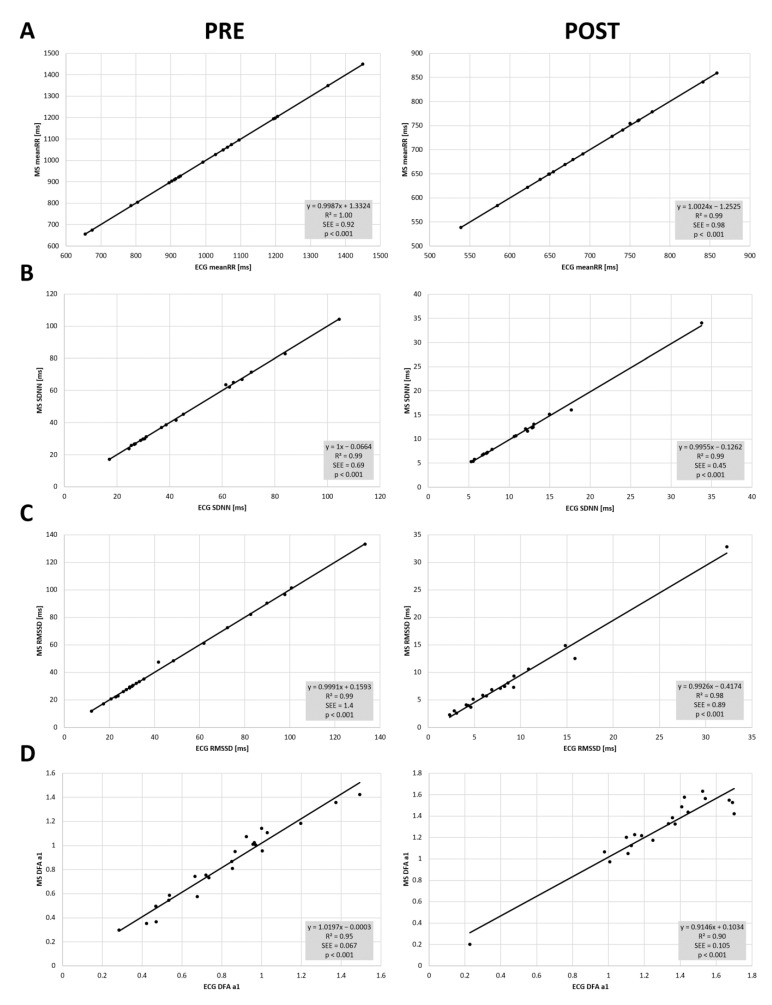
Regression plots for the comparison of the 12 channel ECG (ECG) and the Movesense Medical sensor (MS) during PRE and POST the incremental exercise test for meanRR (**A**), SDNN (**B**), RMSSD (**C**), and DFA a1 (**D**). Slope, coefficient of determination (R^2^), standard error of estimate (SEE), and *p* value shown in the bottom right of each plot.

**Figure 4:**
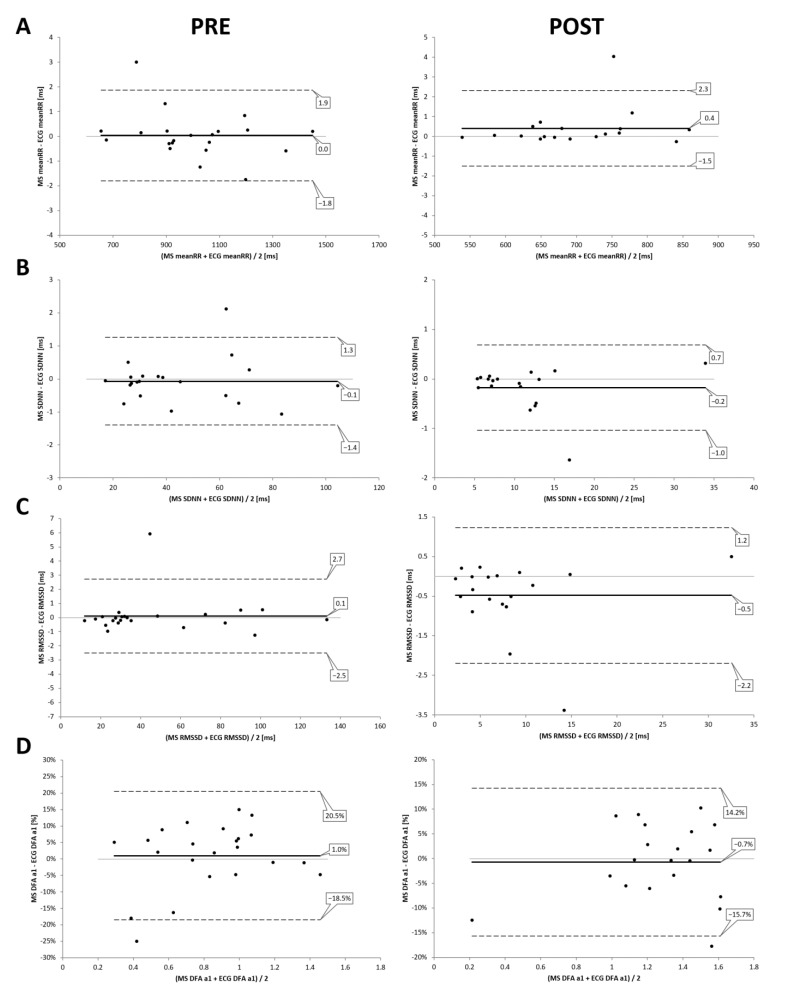
Bland–Altman analysis for the comparison of the 12 channel ECG (ECG) and the Movesense Medical sensor (MS) during PRE and POST the incremental exercise test for meanRR (**A**), SDNN (**B**), RMSSD (**C**), and DFA a1 (**D**). Center solid line in each plot represents the mean bias (difference) between each paired value as absolute time (ms) or relative differential (difference/mean × 100) in the case of DFA a1. The top and bottom dashed lines are 1.96 standard deviations from the mean difference.

High correlations between the two devices could be found in resting state conditions (PRE and POST incremental exercise testing) with meanRR (PRE: r = 1.00, R^2^ = 1.00, *p* < 0.001; POST: r = 0.99, R^2^ = 0.99, *p* < 0.001), SDNN (PRE: r = 0.99, R^2^ = 0.99, *p* < 0.001; POST: r = 0.99, R^2^ = 0.99, *p* < 0.001), RMSSD (PRE: r = 0.99, R^2^ = 0.99, *p* < 0.001; POST: r = 0.99, R^2^ = 0.98, *p* < 0.001) and DFA a1 (PRE: r = 0.97, R^2^ = 0.95, *p* < 0.001; POST: r = 0.95, R^2^ = 0.90, *p* < 0.001) (see Figure 3). Comparing the two devices, t testing revealed statistical differences only for RMSSD POST exercise, but with small effect size (PRE—meanRR: *p* = 0.86, d = 0.0; SDNN: *p* = 0.64, d = 0.0; RMSSD: *p* = 0.68, d = 0.0; DFA a1: *p* = 0.27, d = 0.0; POST—meanRR: *p* = 0.09, d = 0.0; SDNN: *p* = 0.10, d = 0.0; RMSSD: *p* = 0.02, d = 0.0; DFA a1: *p* = 0.79, d = 0.0). Mean and standard deviation are presented in Table 1. Bland–Altman analysis for meanRR showed a mean difference of 0.0 ms (±0.9) with upper and lower LOA of 1.9 to −1.8 ms in PRE, and mean difference of 0.4 ms (±0.9) with upper and lower limits of 2.3 to −1.5 ms in POST. Please see detailed Bland–Altmann plot analysis for meanRR, SDNN, RMSSD, and DFA a1 in PRE and POST exercise in Figure 4.

### 3.2. Incremental Exercise Analysis

**Figure 5:**
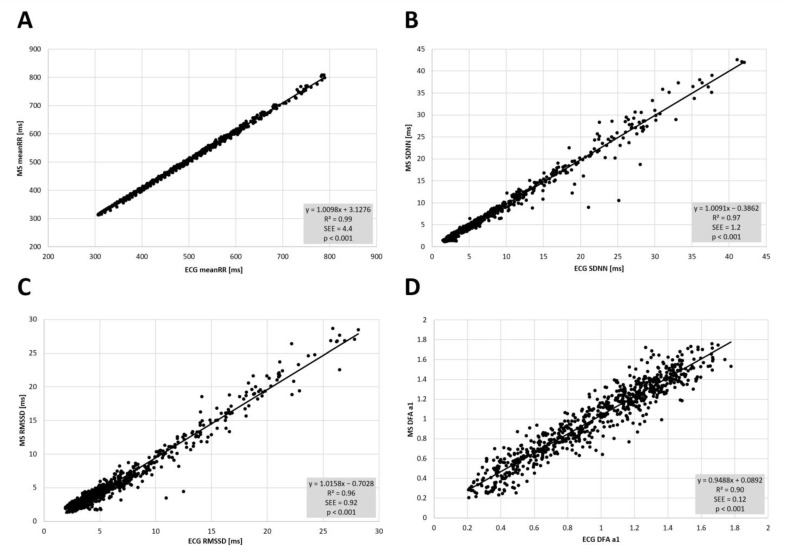
Regression plots for the comparison of the 12 channel ECG (ECG) and the Movesense Medical sensor (MS) during the incremental exercise test for meanRR (**A**), SDNN (**B**), RMSSD (**C**), and DFA a1 (**D**). Slope, coefficient of determination (R^2^), standard error of estimate (SEE), and *p* value are shown in the bottom right of each plot.

**Figure 6:**
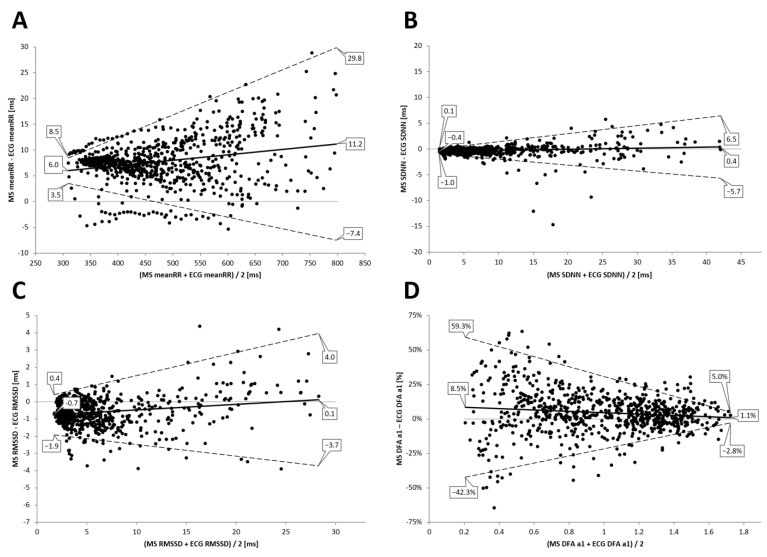
Bland–Altman analysis for the comparison of the 12 channel ECG (ECG) and the Movesense Medical sensor (MS) during the incremental exercise test for meanRR (**A**), SDNN (**B**), RMSSD, (**C**) and DFA a1 (**D**). Center solid line in each plot represents the mean bias (difference) between each paired value as absolute time (ms) or relative differential (difference/mean × 100) in the case of DFA a1. The top and bottom dashed lines are 1.96 standard deviations from the mean difference.

High correlations between the two devices could be found during the incremental exercise test for meanRR (r = 0.99, R^2^ = 0.99, *p* < 0.001), SDNN (r = 0.98, R^2^ = 0.97), RMSSD (r = 0.98, R^2^ = 0.96, *p* < 0.001), and DFA a1 (r = 0.95, R^2^ = 0.90, *p* < 0.001) (see Figure 5 and detailed stats in Table 2). Bland–Altman analysis for meanRR showed a statistically significant mean difference of 11.2 (±9.3) to 6.0 ms (±1.3) with upper and lower LOA of 29.8 to −7.4 ms during low intensity exercise and of 8.5 to 3.5 ms during high intensity exercise. Detailed Bland–Altmann plot analysis for meanRR, SDNN, RMSSD, and DFA a1 took place during the exercise in Figure 6.

## 4. Discussion

The aim of this study was to determine whether the Movesense Medical sensor chest belt device can provide accurate ECG recordings for bias free HRV computations, similar to a reference 12 channel ECG. This was evaluated by comparing RR intervals, linear and non-linear HRV index properties both at rest and during and after incremental cycling exercise. Results showed high agreement between the chest strap sensor and the reference ECG in terms of meanRR, SDNN, and RMSSD measurements, as well as the non-linear index DFA a1. Resting and post-exercise linear HRV values had similar means with generally no relevant divergence. Although different from a statistical standpoint as measured by Wilcoxon testing with Hodges–Lehmann shift measurements, linear HRV comparison differences during the exercise ramp were minimal. Based on the results presented, the Movesense module can be used in lieu of a reference ECG for the computation of HRV indexes. HR would also be expected to track very closely, even through an exercise ramp, as this is a simple calculation based on the RR interval time. With the additional ability of being able to identify both artifact types and differentiate between noise and arrhythmia through the single channel ECG usage, the Movesense Medical module represents an attractive device for mobile ECG recording in lab-based or remote field-application in health and disease.

Examination of individual HRV index and testing conditions were consistent with other reports concerning current HRV monitoring devices, such as the Polar H10 [65]. For linear HRV measures during PRE and POST, the Movesense Medical device derived RR intervals, SDNN and RMSSD, correlated highly to those derived from the reference unit, with Pearsons’s r near 1.0. Bland–Altman analysis also being favorable with minimal bias varying between 0 and 1 ms. LOA were narrow, with values between 1 and 3 ms. During the course of the exercise ramp, the above-mentioned parameters continued to show excellent correlation with Pearson’s r of 0.98 to 0.99. Bland–Altman analysis did show a small difference of about 10 ms in meanRR, but only 1 ms in both SDNN and RMSSD during the incremental exercise. Given the possibility of a slight differential between the device measurement window timing synchronization, this is not unexpected. 

The results of DFA a1 comparisons between devices were of interest. The DFA a1 index is based on fractal correlation properties of the cardiac beat pattern and has been studied as both a tool for exercise intensity assessment, physiologic threshold determination, as well as mortality and cardiovascular risk assessment in different populations [50,51]. Since this index is based on actual patterns of RR intervals over time, comparison of mean or median RR intervals may not predict correlation pattern accuracy. Past studies looking at the effect of introducing slight RR timing errors illustrate the potential for error introduction into this index [66]. In regard to DFA a1, resting and post-exercise differences were negligible at about 1% with LOA between 10 to 20% and Pearson’s r of 0.97 for resting and 0.95 for post-exercise recordings. During incremental exercise, the present results show a much larger Bland–Altman bias, wider LOA, and slightly weaker correlations between the reference ECG and the Movesense Medical sensor. During the exercise ramp, DFA a1 bias was proportional, with amounts approaching 9% and LOA near 40 to 60% at very low index values during high exercise intensity. Pearson’s r was still 0.95 during the exercise ramp, showing high correlation. 

**Figure 7:**
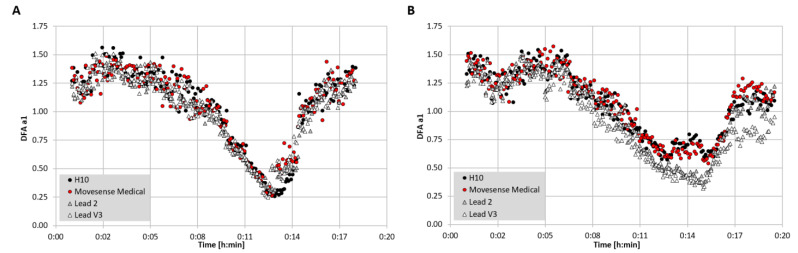
DFA a1 over time during an incremental cycling ramp until voluntary exhaustion with two participants wearing a 12 channel ECG, a Polar H10 chest belt, and a Movesense Medical sensor single channel ECG chest belt. (**A**) One participant with similar DFA a1 seen in both chest belt devices and lead 2 and lead V3 of the 12 channel ECG. (**B**) One participant with discrepancy between DFA a1 in both chest belt devices compared to lead 2 and V3 of the 12 channel ECG. All data were analyzed with Kubios HRV Premium software Version 3.5 (“time varying” option: window width of 2 min, grid interval of 5 s).

In terms of the validation analysis presented, an interesting theoretical question is whether a chest belt derived cardiac electrical waveform is equivalent to one taken from a 12 channel ECG. Although standard lead 2 is felt to be similar to the signal taken from belt recordings across the chest, it is not identical [39]. In addition to QRS waveform morphology, signal strength also appears to play a role in RR timing measurement. Given that this study is comparing both device and lead placement, pinpointing the exact reason for data discrepancy is complex (see also discussion of Weippert et al., [67]). As an example case, a comparison between 3 recording units, including two chest belt devices (Polar H10, Movesense Medical sensor) and a reference 12 channel ECG, may help clarify this issue (Figure 7). When performing an exercise ramp, the measurement of DFA a1 does not appear to show much difference between both chest belt devices and lead 2 or lead V3 of the reference ECG in one participant. However, in another participant, there is a deviation between both chest belt devices and both reference ECG leads. This inhomogeneity may stem from individual variation in the cardiac axis, leading to subtle ECG waveform changes depending on where the signal was sampled [39].

**Figure 8:**
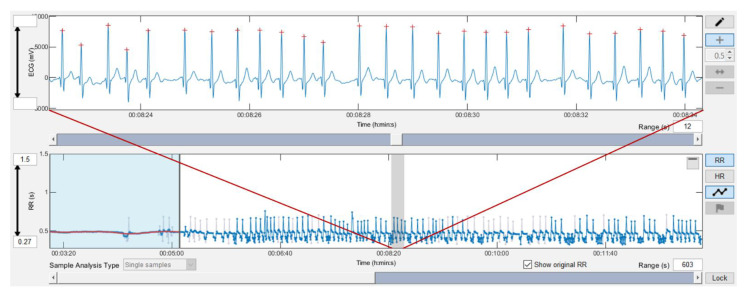
RR interval data during low intensity cycling exercise in one participant recorded with the Movesense Medical chest belt device. The single channel ECG showed excellent waveform quality but frequent atrial premature beats with runs of sustained atrial fibrillation. The data were analyzed with Kubios HRV Premium software Version 3.5.

Since high quality RR interval detection is already available in the latest consumer devices such as the Polar H10 [65], are there reasons to consider further alternatives? Manual identification and correction of noise distorted signals could help keep artifact levels below critical thresholds, thereby minimizing loss of participant- or patient-related data. However, more important from the participant’s health standpoint is the differentiation between an RR series with abundant artifact due to noise or one due to cardiac arrythmia. For example, it has become increasingly recognized that atrial fibrillation occurs more commonly in endurance athletes and can be preceded by frequent atrial premature complexes or atrial tachycardias [68,69]. This is best demonstrated by a case of a previously healthy triathlete undergoing evaluation by our group for a future study. RR recording with a Polar H10 was done concurrently with the Movesense Medical device. Inspection of the RR series from the H10 showed a large degree of artifact, felt to be related to poor chest belt contact due to movement artefacts. However, inspection of the Movesense Medical single channel ECG showed excellent waveform quality but frequent atrial premature beats with runs of sustained atrial fibrillation (see Figure 8). This discovery of a potentially life-threatening arrythmia in a previously healthy individual certainly underscores the need for insight into the nature of RR artifacts and makes a compelling justification for ECG belt usage in both general and athletic populations.

## 5. Limitations and Future Directions 

The sample rates of the ECG reference device and the Movesense Medical sensor were similar but not identical. Most sources recommend device sample rates to be in excess of 250 Hz, with little change expected in the ECG waveform between 500 and 512 Hz [70]. Future investigations should assess the validity of the Movesense Medical sensor during other types of activity or exercise (e.g., impact locomotor activities like walking and running), due to potential influences of substantial movement of the torso and occasionally high force impacts [35,71], extending the application in lab-based and remote settings. In addition, other factors such as sex differences (electrode positioning, skin characteristics), influence of activity or performance level (ventricular size or mass, subcutaneous fat), and the influence of cardiac disease pathology may affect the QRS complex and could interfere with the precise identification of the R wave for HRV analysis [35,72,73,74]. Therefore, validation studies with other populations may be warranted to address these potential issues.

## 6. Conclusions and Practical Implications

HRV measurements derived from the Movesense Medical sensor correspond strongly with a reference ECG in terms of meanRR, SDNN, RMSSD, and the non-linear index DFA a1. It is unreasonable to expect perfect concordance between devices recording electrical signals through differing cardiac lead placements. A major benefit of having an ECG waveform recording is identification of potentially life-threatening arrythmias as well as beat recovery in noisy signals. The Movesense Medical device represents a significant advance in both a HR and HRV recording device in a chest belt form factor for lab-based or remote field-application in health and disease.

## Figures and Tables

**Table 1:** Mean and standard deviation (SD) for the comparison of the 12 channel ECG (ECG) and the Movesense Medical sensor (MS) during PRE and POST the incremental exercise test for meanRR, SDNN, RMSSD, and DFA a1 during PRE and POST condition. * denotes statistical differences in comparison of the two devices with *p* < 0.05.

		meanRR [ms]		SDNN [ms]		RMSSD [ms]		DFA a1	
		ECG	MS	ECG	MS	ECG	MS	ECG	MS
PRE	Mean	1004.2	1004.2	45.0	44.9	47.7	47.8	0.82	0.84
	SD	197.8	197.5	22.6	22.6	31.8	31.8	0.29	0.30
POST	Mean	699.5	699.9	11.3	11.1	8.5	8.0 *	1.27	1.27
	SD	82.2	82.4	6.4	6.4	6.5	6.5	0.32	0.31

**Table 2:** Mean, standard deviation (SD), Median, Minimum, Maximum, adjusted median difference (AMD) for the comparison of the 12 channel ECG (ECG) and the Movesense Medical sensor (MS) according to Hodges Lehmann method (*p*-value estimated by Wilcoxon paired testing), Pearson’s r and Standard Estimate of Error (SEE) from paired data for meanRR, SDNN, RMSSD, and DFA a1 during the incremental exercise.

	meanRR [ms]		SDNN [ms]		RMSSD [ms]		DFA a1	
	ECG	MS	ECG	MS	ECG	MS	ECG	MS
Mean	473.3	481.1	8.1	7.8	6.4	5.8	0.96	1.00
SD	106.4	107.5	7.6	7.8	5.0	5.2	0.37	0.37
Median	455.7	462.1	5.2	4.8	4.5	3.8	0.99	1.04
Maximum	789.4	808.2	42.0	42.6	28.1	28.6	1.77	1.75
Minimum	306.0	313.3	1.4	1.1	1.9	1.3	0.20	0.20
AMD (Wilcoxon)	-	7.68 (*p* < 0.001)	-	0.27 (*p* < 0.001)	-	0.56 (*p* < 0.001)	-	0.04 (*p* < 0.001)
Pearson’s r	-	0.99	-	0.98	-	0.98	-	0.95
SEE	-	4.40	-	1.16	-	0.92	-	0.12

## Data Availability

The datasets analyzed during the current study are available from the corresponding author on reasonable request.

## Abbreviations

DFA a1	short-term scaling exponent of Detrended Fluctuation Analysis
HR	Heart rate
HRV	Heart rate variability
ECG	electrocardiogram
LOA	Bland Altman limits of agreement
R^2^	coefficient of determination
RMSSD	square root of the mean squared differences of the successive RR intervals
RR interval	the time between two successive R-waves of the QRS signal on the ECG
SDNN	standard deviation of RR intervals
SEE	standard error of estimate
V˙O_2MAX_	maximal oxygen consumption
